# The influence mechanism of micropore spatial distribution form on the methane desorption capability in selected Chinese coals

**DOI:** 10.1371/journal.pone.0348714

**Published:** 2026-05-14

**Authors:** Xiaomin Liang, Liankun Zhang, Bo Yin, Hao Guo, Yuhao Chen, Junqing Guo, Bin Zhang

**Affiliations:** 1 School of Resoeurcs and Environmental Engineering, Inner Mongolia University of Technology, Hohhot, China; 2 Jiangsu Branch Kongzhuang Coal Mine, Shanghai Datun Energy Co., Ltd, Xuzhou, China; 3 Key Laboratory of In-situ Property Improving Mining of Ministry of Education, Taiyuan University of Technology, Taiyuan, China; Linköping University: Linkopings universitet, SWEDEN

## Abstract

This study quantitatively investigates the pore structure of coals with different degrees of metamorphism to reveal the spatial distribution forms of micropores and their influence on methane desorption efficiency. The results show that, as coal degrees of metamorphism, the development degree, specific surface area, and pore volume of micropores first decrease and then increase, while mesopores first increase and then decrease, and macropores decrease. The connectivity of macropores declines with increasing coal degrees of metamorphism. Micropores dominate the specific surface area (>96.5%), whereas macropores contribute the most to total pore volume (>45.2%). The study further demonstrates that attached micropores, primarily found in lignite, enable methane to desorb directly into connected seepage channels, resulting in the highest desorption efficiency. Networked micropore clusters, prevalent in coking coal, hinder gas diffusion due to complex pathways, leading to moderate desorption efficiency. In anthracite, isolated micropores impede methane release, producing the lowest desorption efficiency. These findings provide mechanistic insights into the relationship between micropore spatial distribution and gas transport, highlighting the novelty of linking pore topology to desorption behavior. They have significant implications for coalbed methane (CBM) extraction and coal mine safety management, providing guidance on optimizing gas recovery and mitigating geological hazards.

## 1. Introduction

Coal is an important fossil energy resource and a major carrier of coalbed methane [1]. During coalification, the organic structure, aromaticity, pore system, and physicochemical properties of coal continuously evolve, which in turn affects gas adsorption/desorption, transport behavior, and combustion-related safety performance [2]. Previous studies have shown that coal structure can be altered by external factors such as thermal or microwave treatment, and that ignition behavior is closely related to coal composition and structural properties [3,4]. However, although the evolution of pore structure with degree of coal metamorphism has been widely studied, the spatial distribution forms of micropores and their mechanistic influence on methane desorption efficiency remain insufficiently understood.

Recently, the testing methods and scope of research on coal pores have advanced significantly [5,6]. However, the diversity, complexity, and heterogeneity have resulted in a lack of a thorough understanding of the pore structure of coal at present [7]. The classification of coal pores is the key to understanding their origin and characteristics [8]. Scholars define pore structures based on their size, connection, genesis, and geometric shape [9]. From the perspective of genesis, the pores in coal can be categorized as primary, secondary, and metamorphic [10]. Pores are characterized as cylindrical, slit, conical, or ink-bottle morphologies based on geometric characteristics [11]. Based on the different diameters, pores are classified into several types such as ultrafine pores, transitional pores, medium-sized pores and large pores [12]. Pore structure of coal can be characterized as open, semi-closed, or closed. From the perspective of the fluid migration mechanism in pores, coal pores can be characterized as seepage pores or adsorption pores [5]. However, most existing studies on coal pore structure have focused on pore size distribution, pore volume, specific surface area, and morphological classifications based on geometry or function [13]. Although these studies have greatly improved the understanding of coal pore systems, they mainly provide static descriptions of pore characteristics and do not adequately explain how the spatial distribution forms of micropores influence methane desorption and transport behavior. In particular, the role of micropore connectivity, accessibility, and diffusion pathways in controlling methane desorption efficiency in coals of different degree of metamorphism remains insufficiently understood. This knowledge gap limits a more mechanistic understanding of coalbed methane recovery and gas migration in coal reservoirs.

The spatial distribution of micropores in coals of different degree of metamorphism significantly influences methane desorption efficiency, with micropore connectivity and diffusion pathways controlling gas transport. Therefore, this study aims to investigate the pore structure characteristics of lignite of Lingdong Coal Mine, coking coal of Shaqu Coal Mine, and anthracite of Sihe Coal Mine, and to reveal the spatial distribution forms of micropores and their influence on methane desorption efficiency. Based on low-temperature CO₂ adsorption (LTCA), low-temperature N₂ adsorption (LTNA), and mercury intrusion porosimetry (MIP), the pore volume, pore size distribution, and specific surface area of pores at different scales were quantitatively characterized. This work provides a mechanistic basis for understanding methane desorption in coal. It supports United Nations Sustainable Development Goals SDG 7 (Affordable and Clean Energy) and SDG 13 (Climate Action) by improving coalbed methane extraction efficiency and mine safety. This promotes cleaner energy use and reduces greenhouse gas emissions from coal mining [14,15].

## 2. Materials and methods

### 2.1. Materials

The lignite, coking coal, and anthracite used in the experiment were respectively taken from the Lingdong, Shaqu, and Sihe Coal Mine. After on-site coal sample collection, it should be sealed to prevent air oxidation from altering the properties. Table 1 shows the industrial analytical findings calculated using the standards GB/T 6948-2008 and GB/T 212-2008 [16]. It is worth noting that the coal type identification is based on comprehensive grade information rather than a single approximate analytical parameter. In Table 1, the analysis of moisture is clearly indicated on an air-dry basis (ad). Therefore, the low moisture value of the lignite sample reflects the inherent moisture remaining after air-drying in the laboratory, rather than the total moisture content of the raw coal as mined.

**Table 1:** The industrial analysis results of three coals^a^.

Coal samples	*R*_o,max_ (%)	Proximate analysis (wt.%)		
moisture, ad	ash yield, ad	volatile matter, daf
lignite	0.55	6.79	30.17	22.34
coking coal	1.47	1.39	8.32	22.31
anthracite	2.86	1.65	5.21	6.12

^a^ad: air dried basis; daf: dry ash-free basis.

Before the experiment, small coal samples were manually divided from the crushed large samples. Powder coal samples (60–80 mesh, < 200 mesh) and 1 cm³ block samples were obtained. The samples were dried to constant weight at 370 K and sealed. All coal particles selected for the experiment came from the same piece of coal, ensuring consistent physical and chemical properties.

### 2.2. Testing and data processing methods

#### 2.2.1. LTCA test.

At a temperature of 273 K, the Autosorb IQ analyzer studied the CO_2_ adsorption properties of samples of coal with particles smaller than 200 mesh, in accordance with the ISO 15901-3:2007 and ISO 15901-2:2006 standards. To prevent contaminants like water and air from altering the coal’s physicochemical properties during the gas adsorption test, degas the sample tube at 403 K for 12 hours [17]. Then input the sample name and weight into the software, set the adsorption temperature and gas, then start the analysis system.

The Dubinin-Radushkevich model was used to compute the micropore specific surface area (*S*_mic_) and volume (*V*_mic_) in coal. The micropore size distribution curve for coal samples ranging from 0.3 to 1.5 nm was calculated using the Nonlocal Density Functional Theory model [18].

#### 2.2.2. LTNA test.

The standards, instruments used, and testing procedures for LTNA were the same as those for LTCA testing. Test the nitrogen adsorption and desorption parameters for coal samples with particle size less than 200 mesh at 77 K [19]. The mesopore volume (*V*_meso_) and specific surface area (*S*_meso_) in coal samples were calculated using the Barrett-Joyner-Halenda and Brunauer-Emmet-Teller models, respectively [20]. The mesopore size distribution curves of coal samples ranging from 1.5 to 36 nm were displayed using the density functional theory model.

#### 2.2.3. MIP test.

According to the GB/T 29171-2023 standard, the AutoPore V 9620 mercury intrusion meter was used to measure macropores in 1 cm³ of bulk coal samples. First, place the sample to be tested into the expansion meter, seal it, and weigh it. Then, dry helium (He) gas is introduced into the sample cell by a stepwise pressurization method. After reaching the maximum pressure, it is reduced to atmospheric pressure, and the expansive agent is taken out for mercury filling. The mercury (Hg) inlet and outlet pressures, as well as the mercury amount, are automatically recorded. Non-wetting liquids can only enter the pores of materials under the pressure. The size at which mercury can effectively enter the pores decreases as the pressure increases [21]. The Washburn formula was used to characterize this process, and coal samples were measured for their macropore size distribution curve, specific surface area (*S*_macro_), and volume (*V*_macro_).


$$P_{r}=\frac{-2\sigma cos\theta }{r}$$ (1)

Where, *r* is the pore diameter, nm; *P*_r_ is the applied pressure, kPa; θ represents the contact angle between mercury and coal, 140°; *σ* is the mercury’s surface tension, 0.48 N/m.

From the perspective of pore grid connectivity, the pores where mercury can be effectively discharged during the mercury removal process are defined as effective pores, and those where mercury is confined within the pores and cannot be discharged are defined as ineffective pores. Therefore, in this paper, the ratio of the total mercury withdrawal to the maximum mercury intake is defined as the effective pore ratio [5], and the effective pore content in the sample is quantitatively analyzed. The effective pore ratio reflects the connectivity of the pores.

This article classifies pores of various sizes into micropores, mesopores, and macropores based on the International Union of Pure and Applied Chemistry standards, with pore diameters of less than 2 nm, between 2 and 50 nm, and higher than 50 nm, respectively. Different techniques for pore structure characterization have inherent limitations. MIP may be affected by high-pressure compression and the ink-bottle effect; CO₂ and N₂ adsorption results rely on model assumptions and may be limited by diffusion effects. To mitigate these biases, we employed a multi-scale complementary approach combining CO₂ adsorption (micropores), N₂ adsorption (mesopores), and MIP (macropores), which allows reliable characterization of pores across different size ranges. Consistency among these methods and agreement with literature trends provide indirect validation of the results. One representative sample per coal of different degrees of metamorphism was analyzed, and each adsorption and MIP measurement was performed once. The high reproducibility and stability of the gas adsorption and MIP methods ensure that the observed trends are meaningful for comparative analysis among degree of coal metamorphism. However, the limited sample size and absence of replicate measurements constrain the statistical generality of the conclusions. Future work will include multiple samples per coal of different degrees of metamorphism and repeated experiments to enhance the statistical robustness of these findings.

## 3. Results and discussion

### 3.1 Pore structure parameters of micropores analysis in three degree of metamorphism of coals based on LTCA test

#### 3.1.1. The variation law of the degree of micropore development as the degree of coal metamorphism increases.

**Fig 1:**
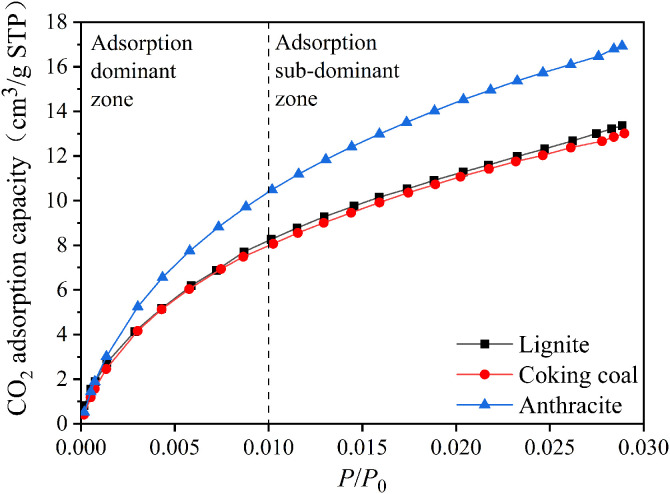
The CO_2_ isothermal adsorption curves of three different degrees of coal metamorphism.

**Fig 2:**
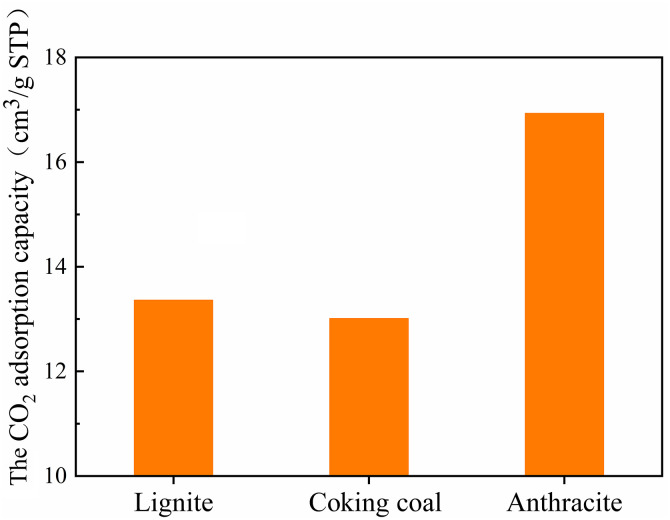
The variation of CO_2_ saturation adsorption capacity of three different degrees of coal metamorphism.

Figure 1 shows the CO_2_ isothermal adsorption curves of three different degrees of coal metamorphism. The three coal samples had a relatively high CO_2_ adsorption rate between 0 and 0.01 relative pressure, which was the adsorption-dominated zone. It has a low CO_2_ adsorption rate between 0.01 and 0.03 relative pressure, which was the adsorption sub-dominant zone. Figure 2 shows the variations in the CO_2_ saturation adsorption capacity of three different degrees of coal metamorphism. Figure 2 shows that the saturated CO_2_ adsorption capacities of lignite, coking coal, and anthracite were 13.36, 13.01, and 16.93 cm^3^/g, respectively, demonstrating that the degree of micropore formation falls and then increases with degree of coal metamorphism.

#### 3.1.2. The variation laws of *S*_mic_ and *V*_mic_ as the degree of coal metamorphism increases.

**Fig 3:**
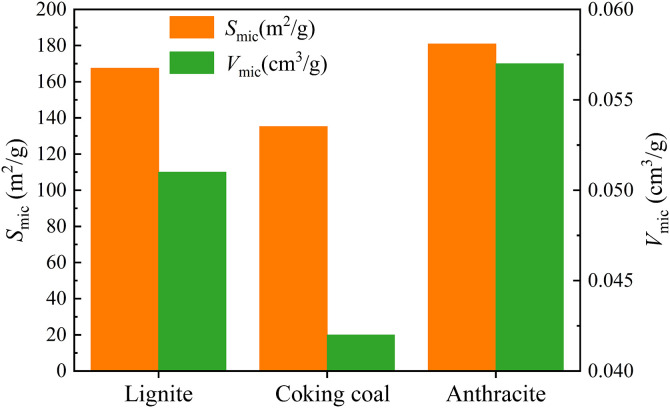
The variation of the *S*_mic_ and *V*_mic_ in three different degrees of coal metamorphism.

Figure 3 shows the variation of the *S*_mic_ and *V*_mic_ in three different degrees of coal metamorphism. Figure 3 shows the *S*_mic_ in lignite, coking coal, and anthracite were 167.455, 135.196, and 180.858 m^2^/g, respectively. The *V*_mic_ were 0.051, 0.042, and 0.057 cm^3^/g, respectively. The *S*_mic_ and *V*_mic_ initially fall and subsequently grow as degree of coal metamorphism increases.

#### 3.1.3. The distribution characteristics of micropore size in three different degrees of coal metamorphism.

**Fig 4:**
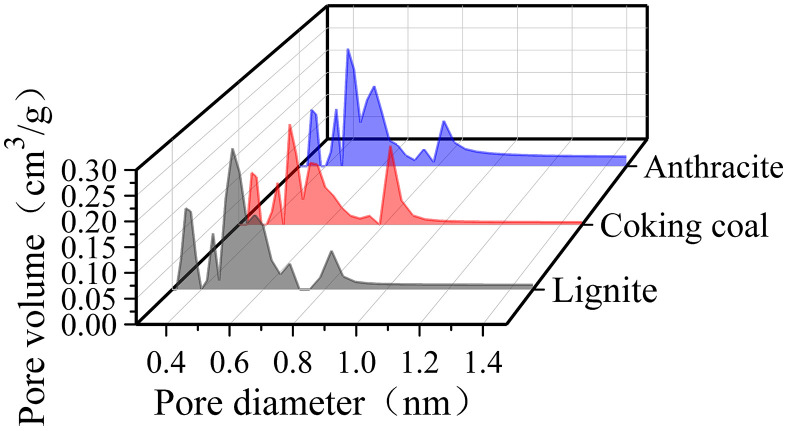
The micropore size distribution curves in three different degrees of coal metamorphism.

Figure 4 shows the micropore size distribution curves in three different degrees of coal metamorphism. The distribution patterns of micropores between 0.3 and 1.5 nm in the three different degrees of coal metamorphism were essentially comparable, as shown in Fig 4.

The micropores volume in the 0.3–0.9 nm has a multi-peak distribution, and it is the primary distribution area for pores in the 0.3–1.5 nm range. When the pore width exceeds 0.9 nm, the pore volume steadily decreases and then stabilizes as the diameter increases. The maximum number of pore diameters representing the dominant pore size is a critical characteristic describing the coal pore structure [22]. The maximum number of pore diameters of lignite, coking coal, and anthracite were 0.5008, 0.4788, and 0.4788 nm, respectively, and the corresponding volumes were 0.2818, 0.2115, and 0.2591 cm^3^/g, respectively.

### 3.2 Pore structure parameters of mesopores analysis in different degrees of coal metamorphism based on LTNA test

#### 3.2.1. The variation law of the degree of mesopore development as the degree of coal metamorphism increases.

**Fig 5:**
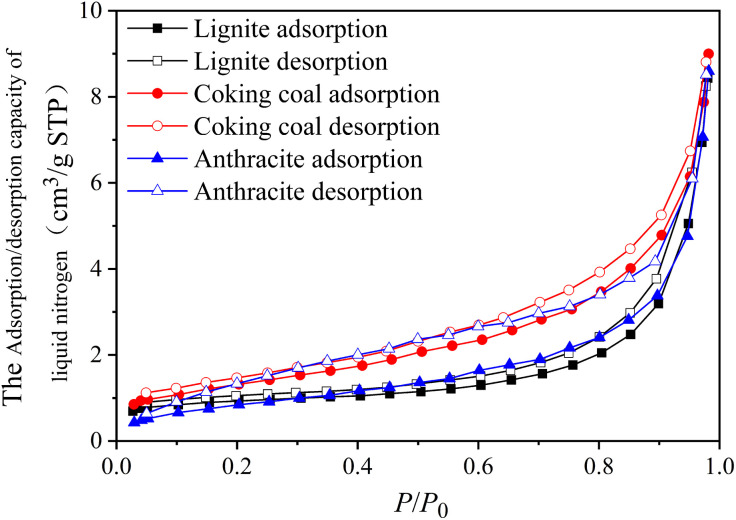
The LTNA isotherms of three different degrees of coal metamorphism.

Figure 5 shows the LTNA isotherms of three different degrees of coal metamorphism. In Fig 5, all the isotherms of the coal samples are separated into three stages. The first is the slow rise stage, which occurs at relative pressures less than 0.4. In this stage, nitrogen experiences single-layer adsorption at low relative pressure on the surface of the pore medium. The adsorption isotherms in this stage protrude upward and rise slowly as the relative pressure increases. The rapid rise stage (0.4 < relative pressure < 0.95), during this medium relative pressure stage, nitrogen adsorption develops from a single layer through multiple layers to capillary condensation, and the adsorption isotherm gradually increases as the relative pressure rises. During the sharp rise stage (relative pressure > 0.95), when the relative pressure approaches 1, nitrogen is mainly adsorbed in the macropores. The nitrogen is difficult to adsorb in macropores, and the adsorption isotherm increases significantly with relative pressure.

According to the IUPAC standards [23], three coal samples’ LTNA isotherms were classified as type IV, and their hysteresis loops resemble type H3, indicating that the pore structures of these coals were highly irregular, mainly consisting of smooth gaps, fractures, and wedge-shaped structures.

**Fig 6:**
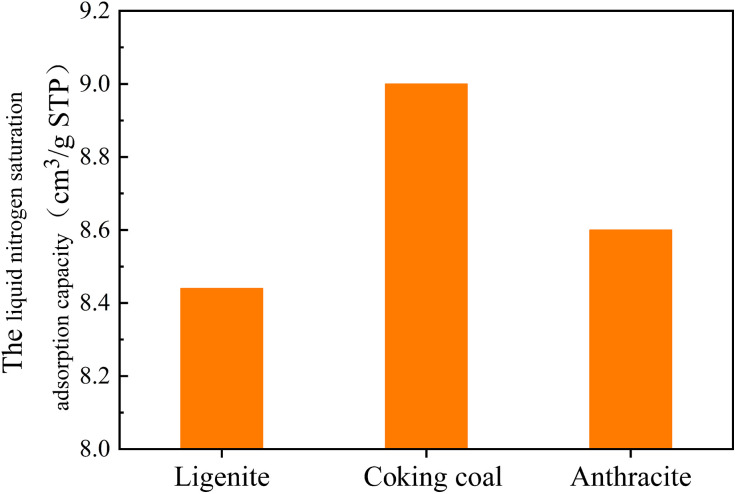
The variation of liquid nitrogen saturation adsorption capacity of three different degrees of coal metamorphism.

Figure 6 shows the variations in the saturated adsorption capacity of liquid nitrogen of three different degrees of coal metamorphism. In Fig 6, the maximum nitrogen adsorption capacities of lignite, coking coal, and anthracite were 8.44, 9.00, and 8.60 m^3^/g, respectively, suggesting that the degree of mesopore development initially increases and subsequently declines as degree of coal metamorphism increases.

#### 3.2.2. The variation laws of *S*_meso_ and *V*_meso_ as the degree of coal metamorphism increases.

**Fig 7:**
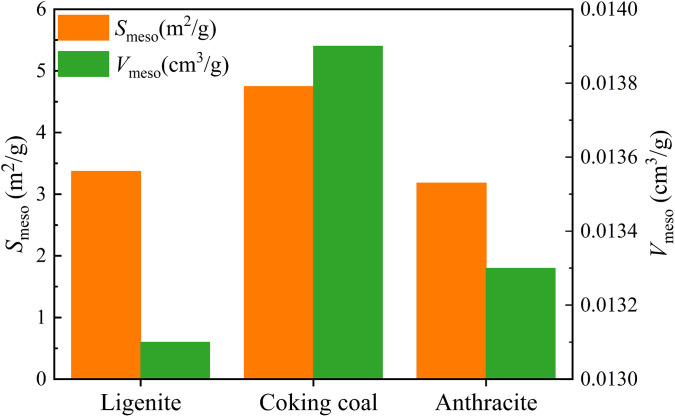
The variations of the *S*_meso_ and *V*_meso_ in three different degrees of coal metamorphism.

Figure 7 shows the variation of the *S*_meso_ and *V*_meso_ in three different degrees of coal metamorphism. In Fig 7, the *S*_meso_ of anthracite, coking coal, and lignite were 3.181, 4.745, and 3.369 m^2^/g, respectively. The *V*_meso_ were 0.0133, 0.0139, and 0.0131 cm³/g, respectively. The *S*_meso_ and *V*_meso_ grow and subsequently decrease as degree of coal metamorphism increases.

#### 3.2.3. The distribution characteristics of mesopore size in three different degrees of coal metamorphism.

**Fig 8:**
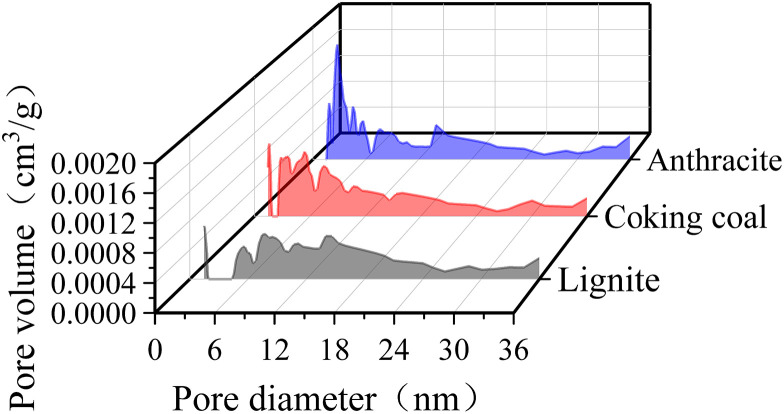
The mesopore size distribution curves in three different degrees of coal metamorphism.

To further understand the fluctuation in the number of mesopore sizes, the mesopore size distribution curves in three different degrees of coal metamorphism, as shown in Fig 8, were obtained by applying the aforementioned data processing method. In Fig 8, the maximum pore diameters of anthracite, coking coal, and lignite were 2.7691, 1.6137, and 1.4748 nm, respectively, and the corresponding pore volumes were 0.0017, 0.0010, and 0.0007 cm^3^/g respectively.

### 3.3 Pore structure parameters analysis of micropores in different degrees of coal metamorphism based on MIP test

#### 3.3.1. The variation law of the degree of macropore development as the degree of coal metamorphism increases.

**Fig 9:**
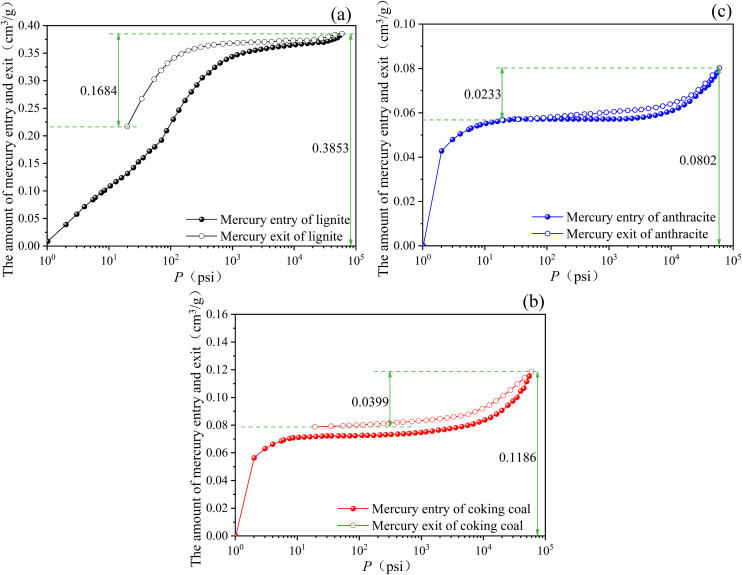
The curves of mercury intake and mercury withdrawal in three different degrees of coal metamorphism. (a) Lignite, (b) Coking coal, (c) Anthracite.

Figure 9 shows the mercury intake and withdrawal curves of three different degrees of coal metamorphism.

In Fig 9, the volume difference between mercury intake and withdrawal in lignite is relatively large, and the hysteresis loop is wide, indicating that mercury in lignite can easily withdraw from numerous open pores, with a high pore connection rate. The volume difference of mercury intake and withdrawal in coking coal is relatively small, and the hysteresis loop is relatively narrow, showing that the majority of the coal sample’s pores are open, but also several semi-open pores. Mercury in coking coal is difficult to extract due to its semi-open pores and poor pore connectivity. The volume difference of mercury intake and withdrawal in anthracite is the smallest, and the hysteresis loop is the smallest, indicating that withdrawing mercury from anthracite is challenging due to the presence of numerous semi-open pores and inadequate pore connections.

**Fig 10:**
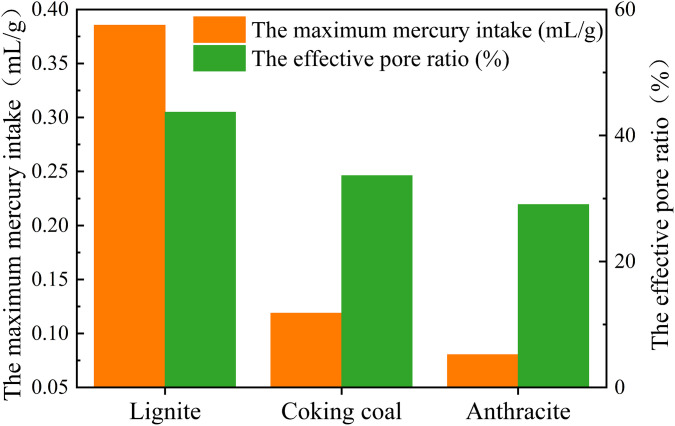
The variation of the maximum mercury intake of three different degrees of coal metamorphism.

Figure 10 shows the variations in the maximum mercury intake of three different degrees of coal metamorphism. In Fig 10, the maximum mercury intake and the effective pore ratio of lignite were 0.3853 mL/g, 43.71% respectively. The maximum mercury intake and the effective pore ratio of coking coal were 0.1186 mL/g, 33.64% respectively. The maximum mercury intake and the effective pore ratio of anthracite were 0.0802 mL/g, 29.05% respectively. It implies that as degree of coal metamorphism increases, the degree of macropore development reduces, as does pore connectivity.

#### 3.3.2. The variation laws of *S*_macro_ and *V*_macro_ as the degree of coal metamorphism increases.

**Fig 11:**
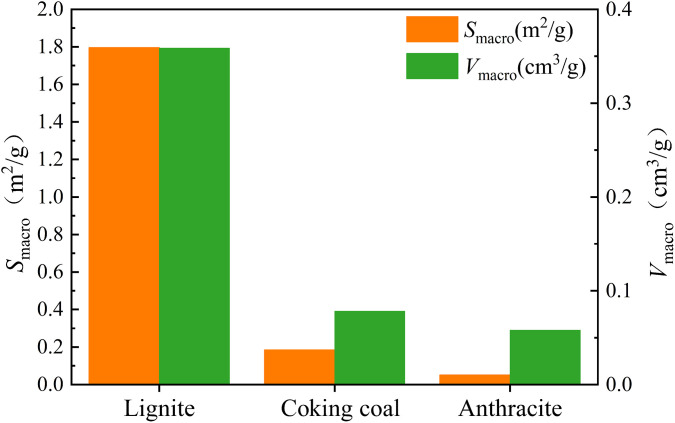
The variations of the *S*_macro_ and *V*_macro_ in three different degrees of coal metamorphism.

Figure 11 shows the variation of *S*_macro_ and *V*_macro_ in three different degrees of coal metamorphism. In Fig 11, the *S*_macro_ of lignite, coking coal, and anthracite were 1.7954, 0.1847, and 0.0511 m^2^/g, respectively. The *V*_macro_ were 0.3587, 0.0782, and 0.0580 cm³/g, respectively. The ongoing condensation of coal structure throughout the coalification process causes the *S*_macro_ and *V*_macro_ to progressively decrease as degree of coal metamorphism rises.

#### 3.3.3. The distribution characteristics of macropore size in three different degrees of coal metamorphism.

**Fig 12:**
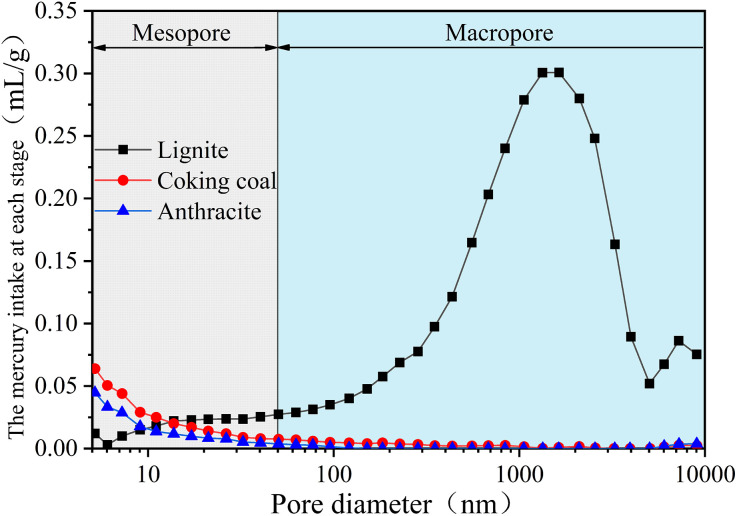
The macropore size distribution curves in three different degrees of coal metamorphism.

Figure 12 shows the macropore size distribution curves in three different degrees of coal metamorphism. In Fig 12, the macropore size distribution curves for the three different degrees of coal metamorphism as a whole show a bimodal feature, located in the mesopore region (5–50 nm) and the macropore region (50–10,000 nm), respectively. The maximal pore size of lignite is around 1050 nm, and the *V*_macro_ of lignite is bigger than the *V*_meso_ because the cumulative mercury intake in the macropore region is substantially higher than that in the mesopore range. Macropores predominate in the pore structure of low degree of metamorphism coal, while mesopores predominate in mid and high degree of metamorphism coal.

## 4. The mechanism of the spatial distribution form of micropores in coal on the desorption capacity of methane

To precisely and fully characterize the morphological properties of pores of different scales in various degrees of coal metamorphism [24], The pore structure characteristics of macropores, mesopores, and micropores of three different degrees of coal metamorphism were determined by summarizing the research findings in the preceding paragraph, as shown in Table 2.

**Table 2:** Pore structure parameters of micropores, mesopores, and macropores in three different degrees of coal metamorphism.

Coal samples	LTCA test				LTNA test				MIP test				Pore connectivity (%)
*V*_mic_ (cm^3^/g)	*V*_mic_ proportion (%)	*S*_mic_ (m^2^/g)		*S*_mic_ Proportion (%)	*V*_meso_ (cm^3^/g)	*V*_meso_ proportion (%)	*S*_meso_ (m^2^/g)	*S*_meso_ Proportion (%)	*V*_macro_ (cm^3^/g)	*V*_macro_ proportion (%)	*S*_macro_ (m^2^/g)	*S*_macro_ Proportion (%)
Lignite	0.051	12.1	167.455	97.0	0.0131	3.1	3.369	2.0	0.3587	84.8%	1.7954	1.0	43.71
Coking coal	0.042	31.3	135.196	96.5	0.0139	10.4	4.745	3.4	0.0782	58.3%	0.1847	0.1	33.64
Anthracite	0.057	44.4	180.858	98.2	0.0133	10.4	3.181	1.7	0.0580	45.2%	0.0511	0.1	29.05

As can be seen from Table 2, the *S*_mic_ and *V*_mic_ from largest to smallest are anthracite, lignite, and coking coal. The *S*_meso_ and *V*_meso_, from largest to smallest, are coking coal, lignite, and anthracite. The *S*_meso_, *V*_meso_, and pore connectivity, from smallest to largest, are anthracite, coking coal, and lignite. Existing research demonstrates that the *S*_mic_ and *V*_mic_ jointly influence the adsorption capacity of methane in coal, with micropore filling being the predominant type of methane occurrence in coal [25]. Methane in coals is mostly found in micropores and adsorption on their surfaces.

The test results show that the *S*_mic_ in low, medium, and high degree of metamorphism coal were 167.455, 135.196, and 180.858 m^2^/g, respectively, and the proportion of micropore volume were 12.1%, 31.3%, and 44.4%, respectively. This means that anthracite and lignite have the highest and lowest methane adsorption capacities. It is consistent with a significant volume of experimental data [26,27]. Further analysis revealed that the *S*_mic_ proportions in low, medium, and high degree of metamorphism coal were 97.0%, 96.5%, and 98.2%, respectively; the *V*_macro_ proportions were 84.8%, 58.3%, and 45.2%, respectively. The proportion of *S*_mic_ in the three different degrees of coal metamorphism all exceeded 96.5%, indicating that the micropores account for the vast bulk of the specific surface area of coal pores. The proportion of *V*_macro_ in the three coal samples is the largest, all exceeding 45.2%. In coal, *V*_macro_ has a significant impact on pore volume composition.

Previous research indicates that low degree of metamorphism coal has a higher fraction of macropore volume than high degree of metamorphism coal [28], resulting in significantly increased methane desorption capacity [29]. However, even if the proportion of macropore volume in low degree of metamorphism coal is higher than 80%, methane still needs to be desorbed from micropores and their surfaces [30], and then enter the percolation pores. This means that in low degree of metamorphism coal, methane has a strong ability to be desorbed and diffused from micropores and their surfaces. While in high degree of metamorphism coal, methane filled in micropores and adsorbed on their surfaces is difficult to be desorbed, diffused, and seep out smoothly. Therefore, the spatial distribution pattern of micropores may be the cause of the property differences among different degrees of coal metamorphism.

**Fig 13:**
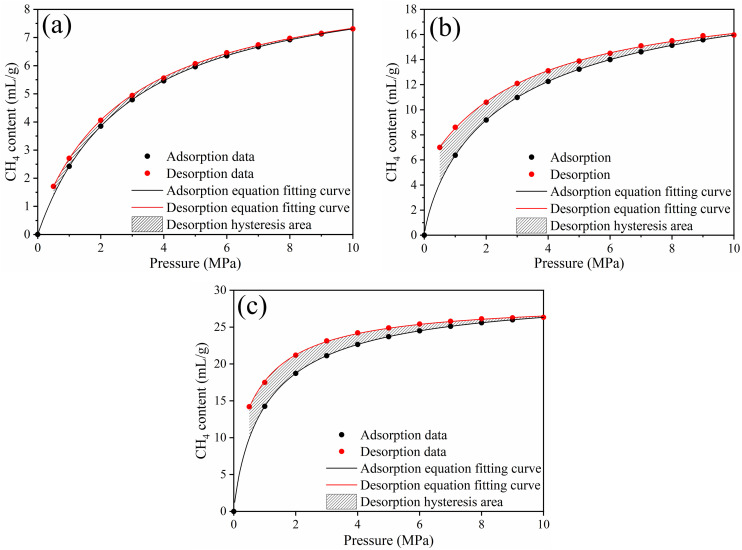
The isothermal adsorption and desorption curves of methane for coals with different degrees of metamorphism. (a) Lignite, (b) Coking coal, (c) Anthracite.

Our previous research results indicate [31]: methane isothermal adsorption–desorption tests were conducted at 313 K with a maximum adsorption pressure of 10 MPa, with equilibrium measured at 1 MPa intervals (10 adsorption equilibrium points). The adsorption equilibrium time was 1800 s, with a standard deviation of 0.008 MPa. Coal samples of 60–80 mesh were then used for variable-pressure desorption tests at equilibrium pressures of 9, 8, 7, 6, 5, 4, 3, 2, 1, and 0.5 MPa, and the test was terminated when the desorption pressure fell below 0.5 MPa. The isothermal adsorption–desorption curves for lignite, coking coal, and anthracite are shown in Fig 13.

To quantify the desorption hysteresis phenomenon, we used the improved desorption hysteresis index (*IHI*). Higher *IHI* values indicate more complex pore structures and more difficult methane desorption. The residual adsorption (*c*) represents the methane content remaining in coal after desorption to near-zero pressure. The theoretical desorption rate (*η*) is the ratio of methane desorbed at the end of the variable-pressure desorption to the methane saturation adsorption amount. The desorption parameters for lignite, coking coal, and anthracite are summarized in Table 3.

**Table 3:** Methane desorption parameters of coals with different degrees of metamorphism.

	Anthracite	Coking coal	lignite
*η*	64.02%	65.00%	96.10%
*c*	7.14 mL/g	5.10 mL/g	0.34 mL/g
*IHI*	31.9%	23.90%	6.47%

From Table 3, it can be seen that the *IHI* decreases in the order of anthracite > coking coal > lignite, while the *c* increases in the same order. The *η* is highest for lignite, followed by coking coal, and lowest for anthracite, indicating that methane desorption becomes more difficult with increasing degree of coal metamorphism.

The microporous properties of coal not only determine the gas storage capacity but also have a significant impact on the desorption efficiency of methane [32]. The methane desorption behavior in coal is strongly influenced by the micropore characteristics [33], including *V*_mic_, *S*_mic_, and the spatial distribution form of micropores. As can be seen from the results of the previous experiments, Lignite has a relatively low *V*_mic_ (0.051 cm³/g) but high macropore connectivity, which facilitates methane diffusion from micropores into larger seepage channels. Consequently, the *η* is highest for lignite (96.10%). In contrast, anthracite has the highest *V*_mic_ (0.057 cm³/g) but most micropores are isolated, resulting in lower effective diffusion paths. This explains its lower desorption efficiency (*η* = 64.02%). Therefore, while a larger *V*_mic_ provides more adsorption sites, its effect on desorption efficiency strongly depends on the connectivity and accessibility of these micropores [34]. *S*_mic_ dominates the coal’s total specific surface area, with values exceeding 96% in all degrees of coal metamorphism. A higher *S*_mic_ correlates with a stronger methane adsorption capacity but also contributes to greater *IHI*, as more methane molecules are adsorbed within narrow micropores that are difficult to release. For example, anthracite exhibits the highest *S*_mic_ and the largest *IHI* (31.9%), reflecting that methane desorption is hindered in tightly packed micropores. In lignite, micropores are mostly attached micropores, connected to macropores, which allows rapid desorption during pressure reduction. In coking coal, micropores form networked clusters, partially interconnected but complex, slowing the diffusion of methane and leading to moderate desorption rates and *IHI* values. In anthracite, micropores are isolated, making methane desorption more difficult despite high *S*_mic_, which explains the highest residual adsorption (*c* = 7.14 mL/g) and lower desorption rate.

In conclusion, micropore characteristics dictate the balance between methane adsorption and desorption. Lignite, with connected micropores and moderate *V*_mic_, allows efficient methane release. Anthracite, despite having large *V*_mic_ and *S*_mic_, shows slow desorption due to isolated micropores. These findings highlight the critical role of the spatial distribution form of micropores in controlling methane recovery efficiency and desorption kinetics.

From a gas transport perspective, attached micropores are distributed on macropore or fracture surfaces, allowing methane to desorb directly into connected seepage channels, significantly shortening the diffusion path. According to Fick’s diffusion law, flux is inversely proportional to the diffusion path length, and shorter paths enhance gas transport rates. Moreover, the connectivity between attached micropores and macropores enables a continuous adsorption–diffusion–seepage process. In contrast, networked micropore clusters have complex, partially closed structures, and isolated micropores lack effective connectivity, both increasing mass transfer resistance and reducing desorption efficiency. At the micropore scale, gas transport may also be controlled by Knudsen diffusion, whose coefficient depends on pore size and geometry, further highlighting the importance of micropore spatial distribution on methane desorption efficiency [35].

**Fig 14:**
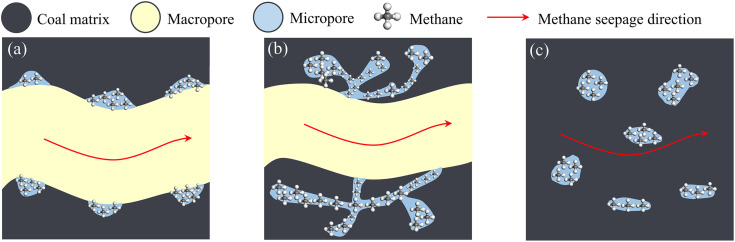
The mechanism of the influence of the spatial distribution form of micropores in coal on the desorption capacity of methane.

Figure 14 illustrates the mechanism of the influence of the spatial distribution form of micropores in coal on the desorption capacity of methane. In Fig 14 (a), low degree of metamorphism coal contains a significantly high amount of *V*_macro_, and most of the micropores are attached to the surface of the macropores. Methane is adsorbed onto the surface of the micropores and fills them. The methane filled and adsorbed in the attached micropores is easily desorbed during the pressure reduction process and directly diffuses into the seepage channels. Because the micropores in low degree of metamorphism coal are mostly connected, the desorption efficiency of methane adsorbed on the surface of the micropores is higher. In Fig 14 (b), the micropores in medium degree of metamorphism coal mostly exist in the form of networked micropore clusters. The micropores are interconnected but intricately complex, and there are some semi-closed pores, which makes it difficult for methane to be effectively extracted even when it is free in the semi-closed pores. In other words, when the pressure decreases, the gas must be desorbed from the micro-pore clusters and then diffuse into the larger fractures through an effective connection network. The existence of the complex network-like micropore clusters reduces the efficiency of methane desorption and diffusion into the main seepage channels. Therefore, the methane desorption efficiency in medium degree of metamorphism coal is usually lower than that in low degree of metamorphism coal. In Fig 14 (c), the proportion of *S*_mic_ and *V*_mic_ in high degree of metamorphism coal is the largest. However, most of the micropores exist independently in the coal matrix, and the methane filled and adsorbed in the independent micropores is difficult to effectively diffuse into the seepage channels. Therefore, the methane in high degree of metamorphism coal is usually difficult to be effectively desorbed.

This study adopts a green engineering approach by optimizing coalbed methane recovery through precise pore-structure characterization using standardized, controlled adsorption methods (CO₂/N₂) that enhance resource efficiency, minimize waste and emissions, support inherently safer processes for disaster prevention, and apply the green chemistry principles of energy efficiency, pollution prevention, and design for safer chemical processes [36,37].

## 5. Conclusions

(1) The specific surface area, pore volume, and degree of development of micropores first decrease and then increase as degree of coal metamorphism rises, whereas mesopores increase first and then decrease, and macropores decrease. The pore connectivity of the macropores decreases as the degree of coal metamorphism increases.
(2) The *S*_mic_ dominates pore specific surface area in coals, while the *V*_macro_ dominates pore volume. The spatial distribution forms of micropores in coal mainly include attached micropores, networked micropore clusters, and independent micropores. These three spatial distribution forms of micropores coexist, but their proportions vary in different degrees of coal metamorphism.
(3) The micropores in low degree of metamorphism coal are mainly attached micropores. The methane filled and adsorbed in the attached micropores can be easily desorbed during the pressure reduction process and directly diffuse into the seepage channels, achieving the highest desorption efficiency of methane. The micropores in medium degree of metamorphism coal mostly exist in the form of networked micropore clusters. The intricate networked micropore clusters reduce the efficiency of methane desorption and diffusion into the main seepage channels, and the methane desorption efficiency is lower than that of attached micropores. The micropores in high degree of metamorphism coal are mainly independent micropores. The methane filled and adsorbed in the independent micropores is difficult to effectively desorb and diffuse into the seepage channels, resulting in the lowest methane desorption efficiency.

Although CO₂ adsorption provides valuable information on micropore size and volume, it cannot directly characterize micropore connectivity or network topology. Future studies will employ high-resolution transmission electron microscopy, scanning electron microscopy, and quantitative image analysis to objectively evaluate micropore spatial forms and their effects on methane desorption. Additionally, extending the analysis to multi-sample validation and pore network modeling will strengthen the generality and applicability of these findings.

The results have practical implications for CBM recovery and coal mine safety, providing guidance for optimizing gas extraction efficiency and mitigating hazards. By linking micropore spatial distribution to desorption efficiency, this study contributes a mechanistic framework that can inform both fundamental research and applied strategies in energy extraction and disaster prevention.

## Supporting information

S1 File Metadata Fig 1–13. (XLS)

## Abbreviations

CBM: coalbed methane
LTCA: low-temperature CO₂ adsorption
LTNA: low-temperature N₂ adsorption
MIP: mercury intrusion porosimetry
*S*_mic_: micropore specific surface area
*V*_mic_: micropore volume
*S*_meso_: mesopore specific surface area
*V*_meso_: mesopore volume
*S*_macro_: macropore specific surface area
*V*_macro_: macropore volume
*IHI*: improved desorption hysteresis index
*c*: residual adsorption
*η*: theoretical desorption rate

## References

[1] Meng J, Lyu C, Wang J, Wang L, Nie B, Lyu Y, et al. Mechanical properties and failure mechanisms of different rank coals at the nanoscale. Fuel. 2023;345:128209. doi: 10.1016/j.fuel.2023.128209

[2] Miroshnichenko D, Kaftan Y, Desna N, Nazarov V, Senkevich I, et al. Dependence of the ignition temperature of coals on their properties. Chem Chem Technol. 2018;12(2):251–7. doi: 10.23939/chcht12.02.251

[3] Malyi E, Chemerinskii M, Golub I, Starovoit M. Thermochemical conversion of coal under microwave radiation. ChChT. 2018;12(4):533–7. doi: 10.23939/chcht12.04.533

[4] Wang H, Shen J, Gao J, Wang W, Zhu L, Gu Y, et al. Cost estimation of Non-CO2 greenhouse gas emissions reduction- a bottom-up analysis of coal-bed methane extraction and utilization in Shanxi, China. Energy. 2024;309:133007. doi: 10.1016/j.energy.2024.133007

[5] Wang K, Guo L, Xu C, Wang W, Yang T, Hu Y. Research on coal reservoir pore structures: progress, current status, and advancing. Nat Resour Res. 2025;34:919–51. doi: 10.1007/s11053-024-10411-8

[6] Liu D, Zhao Z, Cai Y, Sun F, Zhou Y. Review on applications of X-ray computed tomography for coal characterization: recent progress and perspectives. Energy Fuels. 2022;36(13):6659–74. doi: 10.1021/acs.energyfuels.2c01147

[7] Liu H, Zhang S, Qiao Y, Xie D, Chang L. Multifractal characterization of pore heterogeneity and water distribution in medium- and high-rank coals via nuclear magnetic resonance. Fractal Fract. 2025;9:290. doi: 10.3390/fractalfract9050290

[8] Yu Y, Meng Z, Li J, Lu Y, Xu C, Wang Y. Experimental study on pore structure and permeability of different rank coals related to temperature. Fuel. 2026;405:136737. doi: 10.1016/j.fuel.2025.136737

[9] Sarkar P, Singh KH, Singh TN, Ghosh R. Pore topology. In: Laboratory characterization of shale: measurement and simulation. Cham: Springer Nature Switzerland; 2025. p. 55–82. doi: 10.1007/978-3-031-82877-5_3

[10] Mou P, Pan J, Niu Q, Wang Z, Li Y, Song D. Coal pores: methods, types, and characteristics. Energy Fuels. 2021;35: 7467–84. doi: 10.1021/acs.energyfuels.1c00344

[11] Zhang K, Zou A, Wang L, Cheng Y, Li W, Liu C. Multiscale morphological and topological characterization of coal microstructure: Insights into the intrinsic structural difference between original and tectonic coals. Fuel. 2022;321:124076. doi: 10.1016/j.fuel.2022.124076

[12] Li M, Zhang K, Meng Z, Wang B. Evolution of thermodynamic behaviors of methane adsorption in variable migration pores of coals with different deformations. Fuel. 2025;396:135346. doi: 10.1016/j.fuel.2025.135346

[13] Hewu L, Lyu XL, Hou C, X Z u o p e n g. Influence mechanisms of dynamic metamorphism on the evolution of micro/nano pore structures in tectonically deformed coals. Coal Geol Explor. 2024;52. doi: 10.12363/issn.1001-1986.24.06.0373

[14] Fegade SL. Green hydrocarbons and fuels from municipal polymer waste co-fed with natural gas using a batch catalytic slurry process. Green Technol Sustain. 2024;2(3):100099. doi: 10.1016/j.grets.2024.100099

[15] Fegade SL. Assessment of greenness of catalytic deoxygenation of crop oil for green diesel production. Clean Circ Bioecon. 2024;8:100091. doi: 10.1016/j.clcb.2024.100091

[16] Liang X, Kang T, Kang J, Li H, Zhu W. Synergistic mechanism of ultrasonic-chemical effects on the CH4 adsorption-desorption and physicochemical properties of jincheng anthracite. ACS Omega. 2022;8(1):1079–87. doi: 10.1021/acsomega.2c06425 36643569 PMC9835157

[17] Ni G, Li S, Rahman S, Xun M, Wang H, Xu Y, et al. Effect of nitric acid on the pore structure and fractal characteristics of coal based on the low-temperature nitrogen adsorption method. Powder Technol. 2020;367:506–16. doi: 10.1016/j.powtec.2020.04.011

[18] Gathitu BB, Chen W-Y, McClure M. Effects of coal interaction with supercritical CO2: physical structure. Ind Eng Chem Res. 2009;48:5024–34. doi: 10.1021/ie9000162

[19] Han M-L, Wei X-L, Zhang J-C, Liu Y, Tang X, Li P, et al. Influence of structural damage on evaluation of microscopic pore structure in marine continental transitional shale of the Southern North China Basin: a method based on the low-temperature N2 adsorption experiment. Pet Sci. 2022;19(1):100–15. doi: 10.1016/j.petsci.2021.10.016

[20] Sun M, Yu B, Hu Q, Chen S, Xia W, Ye R. Nanoscale pore characteristics of the Lower Cambrian Niutitang Formation Shale: a case study from Well Yuke #1 in the Southeast of Chongqing, China. Int J Coal Geol. 2016;154–155:16–29. doi: 10.1016/j.coal.2015.11.015

[21] Liu S, Liang Y, Sang S, Wang H, Wang W, Sun J, et al. application of mercury intrusion porosimetry in coal pore structure characterization: conformance effect and compression effect correction. Energies. 2025;18(12):3185. doi: 10.3390/en18123185

[22] Li Z, Ren T, Li X, Cheng Y, He X, Lin J, et al. Full-scale pore structure characterization of different rank coals and its impact on gas adsorption capacity: a theoretical model and experimental study. Energy. 2023;277:127621. doi: 10.1016/j.energy.2023.127621

[23] Wang Z, Li Z, Zhang S, Zhang X. Microstructure changes induced by adsorption/desorption of low rank coals and its desorption hysteresis mechanism. Fuel. 2024;357:129804. doi: 10.1016/j.fuel.2023.129804

[24] Zhou G, Zhao M, Liu Z, Liu Y, Chen J, Ma H, et al. Experimental investigation on the pore structure and gas permeation modification of multi-stage coal treated by different extraction agents. Powder Technol. 2025;466:121467. doi: 10.1016/j.powtec.2025.121467

[25] Wang L, Wu S, Han S, Hu B, Wang Q, Zhang K, et al. Fractal analysis of coal pore structure based on low-pressure gas adsorption and its influence on methane adsorption capacity: a perspective from micropore filling model. Energy Fuels. 2024;38(5):4031–46. doi: 10.1021/acs.energyfuels.3c04724

[26] Jia J, Song H, Jia P. Molecular simulation of methane adsorption properties of coal samples with different coal rank superposition states. ACS Omega. 2023;8(3):3461–9. doi: 10.1021/acsomega.2c07471 36713738 PMC9878655

[27] Yue J, Li Y, Shi B, Zhang M, Xu J, Lou Z. Adsorption preference sites and mechanism of coal with different degrees of metamorphism. Langmuir. 2025;41(31):21190–203. doi: 10.1021/acs.langmuir.5c03211 40739749

[28] Jian K, Fu X, Ding Y, Wang H, Li T. Characteristics of pores and methane adsorption of low-rank coal in China. J Nat Gas Sci Eng. 2015;27: 207–18. doi: 10.1016/j.jngse.2015.08.052

[29] Qingqing W, Yanjun M, Taotao Y a, Liu Z, Xu G, Li K, et al. Differences in the adsorption/desorption characteristics of coal reservoirs with different coal ranks and their effects on the reservoir productivity. mtdzykt. 2023;51:66–77. doi: 10.12363/issn.1001-1986.22.10.0816

[30] Chu P, Xie H, Li C, Liu Q, Lu Z, Lu J. Mechanism of desorption hysteresis in coalbed methane: Insights from microscopic pore properties and adsorption theory. Phys Fluids. 2024;36(1). doi: 10.1063/5.0184321

[31] Liang X, Kang T, Kang J, Zhang X, Zhang L, Li H, et al. Experimental study of influence of natural organic solvent limonene on methane adsorption–desorption behaviors of selected rank coals. Energy. 2024;291:130491. doi: 10.1016/j.energy.2024.130491

[32] Yu Z, Bai J, Yang J, Han L. Influence of pore structure and basic coal properties on gas efficient desorption capacity in coal. ACS Omega. 2025;10(17):17504–14. doi: 10.1021/acsomega.4c10950 40352499 PMC12059927

[33] Liu J, Ren B, Wang C. Pore structure characteristics of middle and low rank coals and their influence on gas desorption characteristics. Coal Sci Technol. 2022;50:153–61. doi: 10.13199/j.cnki.cst.2021-0775

[34] Zhou D, Liu Z, Feng Z, Shen Y. Accessibility of methane at micro-pore passage and its effect on the methane desorption in coal. J China Coal Soc. 2019;44: 2797–802. doi: 10.13225/j.cnki.jccs.2018.1361

[35] Wang K, Guo L, Xu C, Wang W, Yang T, Hu Y, et al. Control mechanisms of methane transport in coal: dominant factors of methane diffusion governed by multi-scale pore structures. Int J Hydrog Energy. 2025;140:315–32. doi: 10.1016/j.ijhydene.2025.05.227

[36] Fegade SL. How green is the approach and chemical. Mater Chem Phys. 2015;154:176. doi: 10.1016/j.matchemphys.2015.01.041

[37] Fegade SL. Red chemistry: principles and applications. Next Sustain. 2024;4:100048. doi: 10.1016/j.nxsust.2024.100048

